# Plasmid Gene Therapy for Monogenic Disorders: Challenges and Perspectives

**DOI:** 10.3390/pharmaceutics17010104

**Published:** 2025-01-14

**Authors:** Marco A. Luís, Marcelo A. D. Goes, Fátima Milhano Santos, Joana Mesquita, Paulo Tavares-Ratado, Cândida Teixeira Tomaz

**Affiliations:** 1 CICS-UBI—Health Sciences Research Centre, University of Beira Interior, 6201-506 Covilhã, Portugal; marco.luis@ubi.pt (M.A.L.); marcelo.goes@ubi.pt (M.A.D.G.); fatima.milhano@quironsalud.es (F.M.S.); joana.mesquita@ubi.pt (J.M.); ptavares@fcsaude.ubi.pt (P.T.-R.); 2 RISE-Health, Faculty of Sciences, University of Beira Interior, 6201-506 Covilhã, Portugal; 3 Departament of Chemistry, Faculty of Sciences, University of Beira Interior, Rua Marquês de Ávila e Bolama, 6201-001 Covilhã, Portugal; 4 Fundación Jiménez Díaz University Hospital Health Research Institute (IIS-FJD), Av. Reyes Católicos, 28040 Madrid, Spain; 5 Department of Medical Sciences, Faculty of Health Sciences, University of Beira Interior, 6201-506 Covilhã, Portugal; 6 Laboratory of Clinical Pathology, Sousa Martins Hospital, Unidade Local de Saúde (ULS) da Guarda, Av. Rainha D. Amélia, 6300-749 Guarda, Portugal

**Keywords:** pDNA, monogenic diseases, non-viral vector, gene therapy

## Abstract

Monogenic disorders are a group of human diseases caused by mutations in single genes. While some disease-altering treatments offer relief and slow the progression of certain conditions, the majority of monogenic disorders still lack effective therapies. In recent years, gene therapy has appeared as a promising approach for addressing genetic disorders. However, despite advancements in gene manipulation tools and delivery systems, several challenges remain unresolved, including inefficient delivery, lack of sustained expression, immunogenicity, toxicity, capacity limitations, genomic integration risks, and limited tissue specificity. This review provides an overview of the plasmid-based gene therapy techniques and delivery methods currently employed for monogenic diseases, highlighting the challenges they face and exploring potential strategies to overcome these barriers.

## 1. Introduction

Rare genetic diseases, or orphan diseases, are a group of mono- and multigenetic diseases that can be hereditary or caused by a sporadic mutation. Due to the low prevalence of these diseases among individuals, these have been neglected by society and the scientific community, hindering the development of novel, innovative, and effective therapies [1]. Rare monogenic disorders are conditions with an individual incidence of less than 0.5‰, each resulting from a single genetic mutation [2]. These disorders can be categorized based on whether they involve mutations in nuclear genes, as it occurs in diseases such as pulmonary cystic fibrosis (CF) [3], or in mitochondrial genes, as in retinitis pigmentosa (RP) [4]. Since the potential economic impact of drugs for rare diseases is significantly lower than that for more common diseases, many of these conditions remain unmet medically. Indeed, few pharmaceutical companies and research groups have pursued the development of treatments for diseases affecting small patient populations [5]. The limited availability of robust animal models and clinical specimens for these disorders has further constrained the progress of preclinical research and the transition of therapies from basic research to clinical applications [6]. Despite these ongoing challenges, new initiatives, such as the European Reference Networks, have brought together the experience of several specialist hospital centers to improve access to diagnosis, information, and care for these patients [7]. In recent years, advances have been made in the research and development of new and effective therapies for rare genetic diseases. These innovative therapies allow for a more targeted approach to create new therapeutic applications, where researchers only need to consider the impact and consequences of one mutated gene. In contrast, polygenic diseases, which involve mutations in multiple genes, present a more complex challenge. The varying types of mutations can affect the progression and severity of the disease, further complicating the development of effective therapies for polygenic rare diseases [8]. Currently, the treatment strategies for these rare monogenic disorders are small-molecule drugs that typically alleviate the symptoms rather than addressing the underlying causes of the diseases [9]. Alpha-1 antitrypsin (AAT) deficiency, CF, and RP are examples of monogenetic diseases lacking targeted treatments, relying only on symptomatic management. The continued administration of augmented plasma injections for AAT deficiency [10], the regular prescription of antibiotics potentially resulting in antibiotic-resistant bacteria in CF [11], and the limited treatment options available for RP which include mainly daily UV-protective glasses, vitamin supplements, and retinoids [12] demonstrate the urgent need for the development of novel therapeutics.

In this context, gene therapy has emerged as a treatment for monogenic diseases, driven by successful outcomes in previously untreatable inborn metabolic orphan disorders, genetic diseases, and cancer [13], thus enabling its potential application in other disorders [14].

## 2. Gene Therapy Strategies

**Figure 1:**
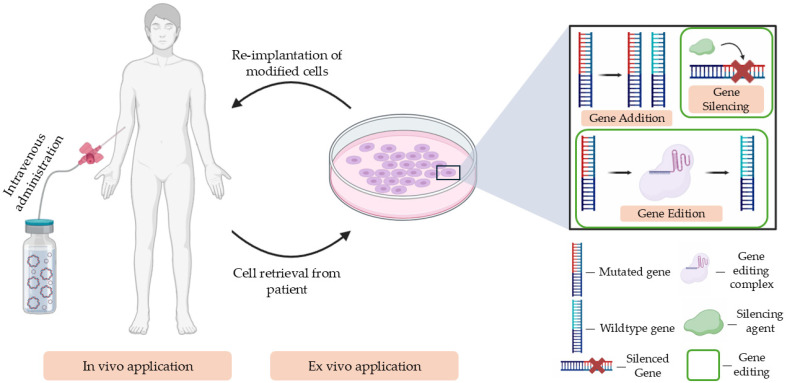
In vivo and ex vivo applications in gene therapy, with the three possible genetic manipulations (gene silencing, gene edition, and gene addition) used in both in vivo and ex vivo therapies.

Gene therapy offers an alternative to conventional therapies for various orphan diseases, using strategies, such as gene silencing, gene edition, and gene addition [15]. These approaches can be applied both in vivo and ex vivo and often involve a combination of different biomolecules and delivery systems (Figure 1).

Gene silencing is a strategy that uses biomolecules such as small interfering ribonucleic acid (RNA) (siRNA) to interfere with and inhibit the production of a specific protein, usually present in abnormal quantities (mutated or not) due to an overexpressed gene [16]. This is achieved through the binding of the siRNA to the RNA-induced silencing complex (RISC), which will maintain the guided strand of the siRNA and direct the complex to the complementary messenger RNA (mRNA) sequence. When the mRNA sequence is recognized by the activated RISC, this specific mRNA is degraded, avoiding the mRNA translation and subsequent protein synthesis in ribosomes [17].

Recently, the application of programmable endonucleases, such as the clustered regularly interspaced palindromic repeats-associated protein 9 (CRISPR/Cas9), in gene editing for gene therapy has gained increased interest. This method offers the potential to directly edit specific genes using a short non-coding guide RNA (gRNA) [18].

In addition, the application of a DNA sequence delivered via viral vectors (adenovirus, retrovirus, or adeno-associated virus (AAV)) has been used for gene addition therapy, a technique based on the transfer of a wild-type transgene that will induce the expression of a functional protein [8] due to its long-term gene expression in humans [19]. Despite AAV being considered to have a lower risk of insertional mutagenesis than other viral vectors, non-viral vectors are considered safer and with lower immunogenicity and cytotoxicity, making them attractive alternatives for gene therapy [20]. Nevertheless, new issues have arisen linked to transient gene expression and the efficiency of transgene delivery of plasmid based non-viral vectors. In fact, plasmids must overcome several obstacles, including degradation by endonucleases in the blood, the blood–brain barrier (BBB), the cell membrane, phagocytosis, and lysosomal degradation before it can finally be taken up into the cell nucleus [21].

**Figure 2:**
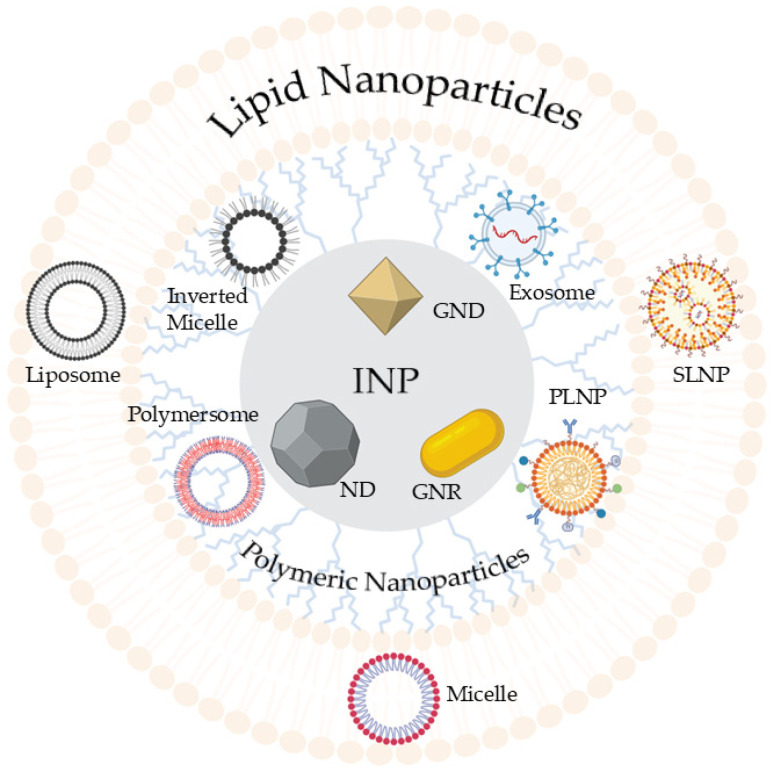
Schematics of gene manipulation tools and delivery systems. Viral and non-viral gene delivery systems (abbreviations: SLNP—solid lipid nanoparticle; PLNP—polymeric lipid nanoparticle; INP—inorganic nanoparticle; ND—nanodiamond; GNR—gold nanorod; GND—gold nanodiamond).

The absence of an appropriate delivery method complicates the process, wherein liposomes, lipid nanoparticles, and/or polymer conjugates can be employed to mask the negative charge and hydrophilicity of the DNA (Figure 2), facilitating the plasmid’s delivery to target cells without degradation [22].

Additionally, plasmid-based vectors overcome transgene size limitations inherent to viral vectors. Engineerable to any size, plasmids accommodate large or multiple transgenes without viral capsid constraints. This versatility makes plasmids ideal for delivering lengthy sequences or complex multigene constructs [19,23]. However, plasmid efficiency is hindered when using a large plasmid, since its transfection can be less efficient and compromise cell viability [24]. Therefore, the choice of vector depends on the type of application, the size of the transgene, the possibility of recurrent administration, and the requirement for prolonged transgene expression [25]. Table 1 shows the comparison of non-viral and viral vectors.

## 3. Non-Viral Gene Therapy

The potential of plasmids as non-viral vectors for gene therapy has been recognized since the nineties [27]. Plasmids offer advantages in terms of production, cost, transportation, and storage, with a considerably longer shelf life compared to viral and RNA-based vectors [28]. Interestingly, the production of viral constructs involves the use of plasmid intermediates to generate viral particles. The modular design of plasmids allows for straightforward molecular cloning, simplifying their manipulation and customization for therapeutic applications. Plasmids have a low integration rate of stable integrants per transfected cell and can be administered multiple times. These key benefits have driven researchers to enhance the safety and efficacy of non-viral DNA vectors [28]. Due to their improved safety profile, plasmids have been employed in numerous clinical trials, including for rare diseases such as monogenic diseases.

In Table 2, we provide an overview of the current applications of plasmid-based gene therapies as a potential alternative to conventional treatments for monogenic diseases.

### 3.1. Ocular Disorders

The systemic route is generally unsuitable for administering gene therapy products to treat posterior segment eye diseases due to the eye’s unique anatomical structure and barrier properties. Instead, the eye is particularly suited for local application, as its compartmentalized structure helps confine the drug to targeted areas, reducing distribution to non-target tissues and minimizing systemic side effects [52]. The effectiveness of gene therapy products in ocular tissues and cells is influenced by these barriers, depending on the chosen administration route. Among these, intravitreal injections have become the preferred method because they deliver the therapeutic products directly into the vitreous humor [53], positioning them close to target areas such as the retina and macula [54]. These characteristics make gene therapy highly promising for improving the prognosis and, potentially, reverse ocular monogenic diseases, which currently lack effective treatments, such as RT, macular telangiectasia type 2 (MTT2), Stargardt disease (STGD) [55].

#### 3.1.1. Retinitis Pigmentosa

RP is a genetically heterogeneous retinopathy characterized by progressive atrophy of the retinal pigment epithelium and apoptosis of photoreceptors resulting in bilateral blindness. This disorder is among the most common and severe types of deteriorating retinal function, which has a prevalence of 1 to 3000 and 1 to 7000, worldwide [56].

The rate of progression of RP varies depending on specific gene mutations and other factors such as inheritance pattern, age of onset, and environmental influences. Research indicates that symptoms may begin in childhood or adulthood, with an average annual visual field loss of 4% to 12% [57]. The severity of vision loss also depends on the genetic subtype; for instance, autosomal dominant RP generally leads to less severe visual impairment, whereas X-linked RP is associated with a more rapid progression and a poorer prognosis [58,59]. Establishing the prognosis for RP is challenging due to its genetic diversity. The wide range of genes, mutations, overlapping conditions, and variability in clinical symptoms associated with the same mutations make it difficult to develop an effective gene therapy treatment for the disorder [56].

Currently, there is no cure for RP, and conventional treatments such antioxidant supplements (retinoids, vitamin A) [60,61], sunlight protection [62], and surgical interventions for ophthalmic comorbidities only aim to slow the progression of the disease.

Given these limited therapeutic options, there is an urgent need to develop novel and personalized treatments, such as gene therapy, to target the genetic causes of RP [15].

Non-syndromic RP is the most common type of the disease, with 56 genes responsible for disease development and nearly 3100 diseases causing mutations, each of which can, possibly, cause variable clinical presentations. As a result, gene therapy ends up being quite compartmentalized, focusing on specific genes and mutations, an approach which often limits its applicability to a broader percentage of cases [56].

Nevertheless, a gene therapy for RP has been approved, albeit exclusively for a limited subset of patients with the RPE65 gene mutation which represents approximately 0.3–1% of all RP cases, among almost 100 involved gene mutations.

Voretigene neparvovec (Luxturna^®^, Novartis) is an AAV serotype 2 vector containing a modified human RPE65 gene encoding retinoid isomerohydrolase, a protein essential for vitamin A metabolism in the visual cycle [63]. In a phase III clinical trial, 31 patients with biallelic RPE65 mutations treated with voretigene neparvovec (Luxturna^®^, Novartis Europharm Limited, Horsham, UK) showed significant visual function improvement, with the benefits lasting three to four years and no serious adverse events reported [52,53]. However, common side effects included ocular inflammation and increased intraocular pressure, potentially linked to the procedure or an immunological response to the viral vector used.

Additionally, the sheer size of approximately 15 kb of the RPE65 poses a challenge for the use of AAV due to their limited packaging capacity of 4.7 kb, making the manufacturing of the vector more complex since the use of different promoters becomes necessary to circumvent this hurdle.

Birch et al. evaluated the use of retinal implants for RP with encapsulated human retinal pigment epithelial cells that were transfected with two separate plasmids encoding ciliary neurotrophic factors (*CNTF*). After 12 months, neither CNTF3 nor CNFT4 implants provided improved therapeutic benefits in the primary outcome variable [64]. Subsequent follow-up of patients who participated in the previously described clinical trial revealed that, over the long term (60–96 months), there was no evidence of efficacy in terms of visual acuity, visual field sensitivity, or OCT measures of retinal structure [30].

More recently, Nolan et al. presented a gene-editing strategy employing CRISPR technology to target the prolyl hydroxylase domain-containing protein gene (*PHD2*) in rod photoreceptors. This study demonstrated an increase in the ratio of glycolysis to mitochondrial oxidation which enhanced rod photoreceptor survival and contributed to the non-cell-autonomous preservation of cone photoreceptors. Furthermore, by applying this strategy to in vivo mouse models of RP, researchers were able to maintain cone viability and rescue rod degeneration, highlighting the potential application of this approach as a gene-editing therapeutic for RP [29].

Different studies have demonstrated the potential of pDNA-based approaches for treating retinal degenerative diseases like RP, showing promising results in terms of gene delivery efficiency, duration of expression, and functional improvements in animal models. However, it is crucial to recognize that these are preclinical studies, and additional research must be conducted to translate these findings into clinical applications for RP patients.

#### 3.1.2. Macular Telangiectasia Type 2

MTT2 is a slowly progressing macular disease where severe cases may involve vision loss due to subretinal neovascularization and retinal pigment epithelium hyperplasia. While it was initially thought to be a vascular disorder, recent findings suggest a neurodegenerative nature, with primary disruption occurring in Müller cells, which are essential for supporting the retinal neurovascular unit and maintaining the blood—retinal barrier [65]. The genetic causes of MTT2 are still not well understood, but a genome-wide linkage analysis has identified a potential association on chromosome 1 at 1q41-42 [66]. Furthermore, mutations in the *PHGDH* gene, which encodes phosphoglycerate dehydrogenase—a key enzyme in the L-serine synthesis—have been found in about 3.2% of cases, indicating a genetic link with the condition [67]. Regarding the MTT2 prevalence, an Australian study estimated it at between 5 and 23 cases per 100,000 people [68]. Currently, there is no effective treatment for MTT2. While some therapeutic approaches use the *CNTF* or control subretinal neovascularization with the anti-vascular endothelial growth factor (VEGF) therapies—commonly used in conditions like diabetic retinopathy [69] and age-related macular degeneration [70]—the results from these strategies fall short of providing a truly effective solution.

Gene therapy efforts using viral vectors in retinal explants face challenges, such as limited carrying capacity for the large genes often needed to treat retinal diseases and low tropism for Müller cells, which are heavily affected in MTT2. To overcome these obstacles, Han et al. (2021) experimented with chimera-dependent adenoviruses to target retinal ganglion cells (RGCs) and Müller cells in both in vivo and ex vivo assays. The results demonstrated successful transduction of Müller cells and RGCs in ex vivo models and of the retinal pigment epithelium in vivo. However, the vectors showed no tropism for photoreceptors in retinal explants and failed to target RGCs or Müller cells in the animal model [71].

Birch et al. developed and evaluated a clinical trial that used retinal implants with encapsulated human retinal pigment epithelial cells that were transfected with two separate *CNTF*-encoding plasmids. After 12 months, neither CNTF3 nor CNFT4 implants provided positive results, revealing that the increase in retinal thickness witness was a dose-depended effect [30].

#### 3.1.3. Stargardt Disease

STGD is another ocular disorder that is focused on macular dystrophy but is caused by a mutation in the *ABCA4* (ATP-binding cassette subfamily A member 4) gene, which encodes for the ATP-binding cassette (ABC) transporter protein. With this mutation, the production of a non-functional ABCA4 protein leads to the accumulation of toxic substances in the photoreceptor cells and peripheral cells, causing cell death. Even though this disease has a very heterogeneous genetic cause, STGD is almost always caused by a reduction in *ABCA4* expression. In terms of prevalence, this disorder is present in approximately 1 to 8000–10,000 individuals and has the peculiarity of being more severe when the onset occurs in the earlier years of life [72]. Currently, STGD is considered untreatable; however, gene therapy has shown itself to be a possible lifeboat for managing this condition. The use of traditional viral vectors, such as AAV, has proven challenging due to the large size of the *ABCA4* gene. Attempts have been made to use dual AAVs and lentiviruses to overcome these limitations, but concerns remain about uncontrolled genome integration, potential oncogenic effects, and low transduction efficiency [32].

To explore a potential therapeutic approach for STGD, Sun et al. (2020) developed two self-assembling nanoparticles for delivering *ABCA4*-encoding pDNA. In their preclinical study, they used a pH-sensitive amino lipid called ECO to form nanoparticles that deliver therapeutic pDNA to *ABCA4* (−/−) mice. This strategy resulted in prolonged gene expression in the outer segments of the photoreceptors and a significant reduction in the accumulation of toxic molecules in the retina compared to controls. Moreover, the nanoparticle delivery system demonstrated excellent safety following subretinal injection [31]. Two years later, Sun et al. improved the self-assembling nanoparticle by adding polyethylene glycol (PEG) molecules, which maintained the original nanoparticle’s capacity for *ABCA4* expression while boosting its therapeutic efficacy, as shown by a further reduction in toxic molecule buildup in the retina. The updated nanoparticle also continued to demonstrate an excellent safety profile, even after repeated retinal injections, suggesting its suitability for ongoing therapeutic applications [32].

### 3.2. Respiratory Disorders

Respiratory disorders include a diverse array of conditions impacting the lungs and respiratory system, often leading to breathing difficulties and potentially life-threatening complications if not properly managed. Among these, genetic disorders like CF and AAT deficiency are particularly concerning due to their severe and chronic symptoms, which can be life-threatening from an early age. These disorders present significant treatment challenges; however, gene therapy has emerged as a promising approach to alleviate or, potentially, reverse their effects, offering hope for more effective long-term management [73]. Many respiratory disorders, like those mentioned above, are monogenic, making them suitable candidates for genetic therapies. However, targeting lung cells can be tricky due to their thick mucus barrier and the need for repeated administration due to constant cell renewal, creating many physical and immunological barriers to effective treatment [74]. Additionally, diagnosing inherited respiratory disorders is challenging due to their broad range of clinical presentations. Accurately correlating the observed phenotypic characteristics with the underlying genetic mutations remains difficult for clinicians, often leading to misdiagnoses. This complexity complicates early intervention and appropriate treatment planning, underscoring the need for improved diagnostic approaches [75].

#### 3.2.1. Cystic Fibrosis

CF is considered one of the most common monogenic diseases, with a general prevalence of 1 in 3500 births, and is associated with symptomatic newborns with low weight gains and respiratory infections [76]. This disease is caused by mutations in the cystic fibrosis transmembrane conductance regulator (*CFTR*) gene, which encodes a crucial anion channel for the transport of chloride and bicarbonate ions across epithelial cell membrane. Of the more than 2000 variants that have been identified in the *CFTR* gene, about 700 have been confirmed as disease-causing. These mutations are categorized into three main types: type I mutations, which result in defective CFTR synthesis due to premature termination codons, type II mutations, which are characterized by impaired folding and trafficking of the CFTR, resulting in few or no CFTR channels at the membrane, and type III, IV, V, and VI mutations, which affect channel gating, conductance, abundance levels, and stability of the CTFR protein, respectively [77].

The most common and prevalent disease-causing mutation in the *CFTR* gene is F508del. This mutation impairs protein processing and causes mislocalization, disrupting the function of chloride channels in mucus-producing cells [78]. Over time, CF causes chronic lung infections and the development of bronchiectasis, driven by excessive inflammation [79]. The prescription of antibiotics and aerosolized systemic steroids are the usual therapeutic strategies to prevent/control infections and reduce inflammation. More recently, CFTR modulators and antibody-based drugs have been approved as an alternative to the standard methods, but they are only capable of controlling already existing symptoms and cannot be used to directly target the root of the disease [77,78,80]. For that objective, gene therapy approaches have been looked upon as an optimistic option for the treatment of CF. Different studies have utilized viral vectors for CF, most notably the work of Wagner et al. that, in 1998, successfully restored CFTR function in clinical trials by delivery of AAV serotype 2-CFTR into maxillary sinuses [81,82]. However, subsequent clinical trials failed to achieve adequate levels of CFTR using this strategy. The CRISPR prime editing strategy represents one of the latest applications of gene editing in mutated *CFTR* cell lines. This innovative tool allows precise nucleotide substitutions, deletions, and insertions. When applied to in vitro *CFTR* models, such as HEK293T and 16HBE cell lines, it enables DNA correction of the mutated *CFTR* gene, restoring proper protein function. These results were further validated using human nasal epithelial cells and patient-derived rectal organoids [36].

Moreover, other viral vectors also face significant challenges, including loss of therapeutic efficacy due to airway cell turnover, insertional mutagenesis, and epigenetic silencing [83].

Additionally, various approaches have focused on non-viral vectors for gene therapy. In 2023, Maze et al. developed a *CFTR*-encoding plasmid for CF gene therapy which demonstrated the ability to produce a fully glycosylated C form of the CFTR protein in human bronchial epithelial cells during preclinical trials, confirming its capability for selective tissue expression [33]. Given the role of inflammation in CF, removing CpG motifs from the plasmid sequence could improve this therapeutic agent, as it has been shown to reduce or eliminate host immune responses [84]. Another potential enhancement is the use of minicircle DNA (mcDNA) instead of pDNA. Due to its smaller size, mcDNA can significantly increase transgene expression compared to pDNA of the same mass, resulting in enhanced and prolonged expression, which could be beneficial for targeting airway cells [37].

In 2015, the first phase 2b clinical trial for CF using plasmid-based gene therapy was conducted, showing beneficial effects after one year of monthly administration compared to placebo [35]. The results indicate that patients with more severe cases experienced double the treatment effect, suggesting stabilization of lung function. However, further studies are needed to enhance the efficacy and consistency of the response [35].

Another gene therapy approach was proposed by Ruan et al., involving pDNA encoding the Cas9 protein, delivered via electroporation or lipofectamine. Unfortunately, neither method proved viable, with the ribonucleoprotein format for Cas9 emerging as the most effective [34].

Sainz-Ramos et al. also developed a cationic lipid-based niosome carrier for gene therapy for CF, proposing an alternative to the use of viral vectors and a more efficient delivery option than the current choice of liposomes. Overall, the study showed that the use of these particles could bring many benefits, reporting a five-fold increase in CFTR protein expression in transfected cells [85].

Due to its higher prevalence, CF has fortunately garnered a broad range of research efforts aimed at developing more effective treatment methodologies, including diverse approaches to gene therapy.

#### 3.2.2. Primary Ciliary Dyskinesia

Primary ciliary dyskinesia (PCD) disease is characterized by a modification in ciliary beat frequency and/or pattern, causing ciliary disfunction, which leads to chronic inflammation and infections as well as reduced efficiency of mucociliary clearance [86]. PCD occurs with a prevalence of 1 in 2200–40,000 individuals, but is more common in consanguineous marriages [87]. PCD is a highly heterogeneous disease linked to over 50 genes with various mutations, many of which have been identified only recently [88]. Among these, mutations in the *DNAH5* gene are the most commonly associated with PCD [89]. The *DNAH5* gene encodes a dynein axonemal heavy chain protein, a motor protein that is crucial for the proper functioning of the dynein arm complex, which drives ciliary motility [90]. Current treatments focus on enhancing mucociliary clearance, reducing inflammation and infection and stabilizing or improving lung function [91]. Although these treatments differ from those used specifically for CF, they share their therapeutic goals. Currently, there have been few direct attempts to use gene therapy for PCD. Ostrowski et al. (2014) tested a hyaluronic acid (HA) pseudotyped lentiviral vector to restore ciliary activity in PCD cells both in vitro and in a mouse model. While the in vitro assays showed promising results in restoring ciliary function, the gene transfer efficiency in the mouse model was significantly compromised by rhinosinusitis, significantly reducing accuracy to around 10% and limiting the overall efficacy of the vector [92]. Although not specific to PCD, Munye et al. demonstrated that using mcDNA complexed with a liposome enabled the expression of *DNAH5* following in vitro transfection of airway cells [37]. In vivo models showed that this approach resulted in lower immunogenicity and a more sustained expression compared to pDNA [84].

#### 3.2.3. AAT Deficiency

AAT deficiency is one of the most common genetic disorders, with a prevalence of 1–5 per 10,000 individuals among European individuals [93]. This disease is caused by a mutation in the *SERPINA1* gene, which is responsible for synthesizing AAT in the liver. The most common mutation is a single amino acid substitution of lysine for glutamic acid at position 342, known as the protease inhibitor phenotype. This missense mutation causes the AAT protein to misfold and form aggregates in the endoplasmic reticulum of hepatocytes, preventing its proper secretion into the plasma [94]. In the lungs, AAT’s primary function is to protect the tissue from neutrophil elastase enzymes. With AAT deficiency, neutrophil numbers increase, and unchecked protease activity leads to structural damage, increased susceptibility to infection, tissue damage, and emphysema [93]. Since 1987, only one treatment for AAT deficiency has been approved: intravenous augmentation therapy with plasma-purified AAT. While this therapy temporarily restores the levels of wild-type AAT levels in circulation, it requires regular and frequent transfusions, typically every 3 to 5 days, to maintain its effectiveness [95]. Due to these limitations, gene therapy is considered a more promising approach for AAT deficiency. Flotte et al. (2011) explored it by using a recombinant AAV serotype 1 vector for gene therapy, showing potential with transgene expression. However, a phase II dose-escalation trial revealed the inefficiency of this strategy, achieving only 3% of the therapeutic target and requiring 100 intramuscular injections, with concerns that higher concentrations could lead to viral aggregation and precipitation. Although the therapy was well-tolerated, it induced a host immune response, indicating the need for alternative delivery methods [94,96]. As a novel approach, Brigham et al. developed a plasmid–liposome complex containing the *SERPINA1* gene, which was administered through aerosolized gene therapy. By applying the plasmid–liposome complex to one nostril and a placebo to the other, researchers observed that the complex demonstrated anti-inflammatory effects, evidenced by a reduction in interleukin-8 levels. Furthermore, AAT expression increased by 33% in the nostril treated with the plasmid DNA compared to the placebo [97]. As one of the latest gene therapy applications for alpha-1 antitrypsin (AAT) deficiency, Shen et al. (2018) employed two CRISPR-based approaches to reduce liver aggregates and increase AAT-M levels in in vivo transgenic mouse models (PiZ). One approach was designed to correct the Z mutation in the *SERPINA1* gene, while the other aimed to reduce AAT expression in hepatocytes. Both strategies successfully achieved their objectives: the expression of the Z-mutated AAT was reduced, while wild-type AAT-M levels were restored to moderate levels [39].

### 3.3. Skin Disorders

The skin functions as the body’s primary protective barrier, accounting for about 15% of total body weight and is composed of three main layers: the epidermis, dermis, and hypodermis. Constantly exposed to environmental threats, it serves as the first line of defense. Its remarkable resilience stems from a complex interplay of various factors, including different cell types, the immune system, and regulatory gene systems. Disruptions in any of these components can have detrimental effects on overall health [98].

Genetic skin diseases, or genodermatoses, are inherited disorders that represent a diverse and significant group of pathologies. Although generally rare, with a prevalence of 1 in 50,000 to 200,000, these conditions often manifest at birth or early in life and can be chronic, severe, and life-threatening. Currently, treatments primarily focus on symptom management; however, as more inherited skin diseases are identified, the development of gene-based therapies for these conditions become increasingly imperative [99].

#### Recessive Dystrophic Epidermolysis Bullosa

Recessive dystrophic epidermolysis bullosa (RDEB) is a rare genetic disorder characterized by extremely fragile skin that blisters and scars easily. It is caused by mutations in the *COL7A1* gene, which encodes type VII collagen, a protein essential for anchoring the epidermis to the dermis. The severity of RDEB depends on the specific *COL7A1* mutation, with an estimated prevalence of two-to-three cases per million live births [100]. Due to the nature of this disease, multiple organs can be affected, potentially leading to other complications such as cardiomyopathy, blindness, and progressive hand contractures.

Advances in molecular genetics and biotechnology have accelerated the development of gene and cell-based regenerative therapies for RDEB, leading progress in clinical trials [101].

Currently, only one gene-therapy-based treatment, beremagene geperpavec (Vyjuvek^®^, Krystal Biotech, Pittsburgh, PA, USA), has been approved for RDEB management [102]. This innovative therapy employs a herpes simplex virus 1 (HSV-1) vector to initiate the transcription of the *COL7A1* gene, restoring COL7 protein production in keratinocytes and fibroblasts, a phenomenon which enhances their development and accelerates healing. COL7 proteins form anchoring fibrils in the extracellular matrix, essential for maintaining dermal–epidermal adherence in the skin. However, as beremagene geperpavec is a relatively new treatment, there is limited information on its long-term risks or side effects. Reported side effects include itching, chills, and redness; however, more concerning is the fact that 10% of patients developed squamous cell carcinoma of the skin [103].

Non-viral gene therapy offers a promising alternative to viral approaches but remains limited by inefficient gene delivery vectors. Poly(β-amino ester)s are notable non-viral candidates due to their versatility, high polymer flexibility, and strong gene transfection performance in both in vitro and in vivo applications. Zeng et al. (2019) developed a non-viral gene therapy for RDEB using nanoparticles made of highly branched poly(b-amino ester) (HPAEs) and minicircle DNA (mcDNA) encoding *COL7A1*. These nanoparticles, featuring DNA-binding components that ensure stability and compactness, achieved higher transfection efficiency in keratinocytes compared to traditional polyethylenimine-based systems, without causing cytotoxicity. Lyophilization further enhanced their transfection performance and preserved their properties for up to one year, making them a promising option for RDEB topical treatment [40]. An in vivo study using RDEB (−/−) mice, which closely resemble the human phenotype with blistering and wound formation, also demonstrated that HPAEs effectively delivers therapeutic pcDNA3.1COL7A1 DNA, enabling type VII collagen restoration in the knockout model. The encouraging in vitro and in vivo outcomes show that branching can greatly improve the gene transfection efficiency of poly(β-amino ester)s, highlighting the potential of HPAEs for clinical use [104,105].

Recently, Berthault et al. has demonstrated a highly efficient ex vivo gene-editing application using CRISPR for RDEB which could serve as a versatile tool for correcting all variants in exon 80 of the *COL7A1* gene in primary RDEB cells. Their study followed a three-stage process. Firstly, three gRNAs targeting the variant c.6508T> were delivered alongside the Cas9 protein, forming a ribonucleoprotein complex. One of the gRNAs achieved a maximum cleavage efficiency of 73% in primary RDEB keratinocytes and fibroblasts. A step of genetic correction using the gRNA, with the highest cleavage activity, was paired with a single-stranded oligodeoxynucleotide donor template to treat RDEB fibroblasts and keratinocytes. This resulted in a 58% genetic correction and a successful rescue of type VII collagen expression. Finally, in the in vivo application, treated three-dimensional skin constructs were grafted onto the mice, resulting in the re-expression and correct localization of type VII collagen. Fibril anchoring at the dermal–epidermal junction was observed five and ten weeks post-grafting. This study highlights the potential of CRISPR-based gene editing as a therapeutic strategy for RDEB [41].

### 3.4. Metabolic Disorders

Inherited metabolic disorders (IMDs) are rare genetic conditions caused by mutations in metabolism-related genes, affecting various biochemical pathways and leading to severe health issues. They make up 20% of rare diseases, with an incidence of 1 in 800 to 2500 live births. IMDs are classified into three subtypes: small molecule defects, energy metabolism disorders, and complex molecule disorders. Clinically, they present a wide range of symptoms that can affect any organ at any stage of life, from prenatal development to adulthood, with some disorders causing acute symptoms and others presenting chronically, often involving multiple organs and, notably, the central nervous system. These disorders are genetically diverse, with changes such as genomic rearrangements, deletions, insertions, or point mutations that disrupt cellular metabolism by altering protein function [106].

#### Niemann–Pick C1

Niemann–Pick disease type C1 (NPC1) is a neurodegenerative metabolic disorder belonging to a group of lysosomal storage diseases, with an incidence of 1.12 per 100,000 live births [107,108]. This disorder is caused by mutations in the *NPC1* gene, which encodes a 142 kDa transmembrane protein responsible for transporting non-esterified cholesterol within lysosomal membranes. A defective NPC1 protein impairs cholesterol and lipid transport, resulting in their accumulation in various tissues, including the central nervous system. This buildup leads to progressive neurodegeneration and hepatosplenomegaly [42]. Depending on the age at the onset, patients may have a short lifespan with symptoms appearing immediately after birth, or they may progress to puberty, but with severe neurological and motor impairments, such as cerebellar ataxia, seizures, and motor dysfunction [109]. The treatment for NPC1 includes drugs commonly used to manage hypercholesterolemia, but in September 2024 the FDA approved two new targeted therapies, arimoclomol (Miplyffa™, Zevra Therapeutics, Celebration, FL, USA) and levacetylleucine (Aqneursa™, IntraBio Inc., London, UK). Miplyffa™ comes as a combination with the enzyme inhibitor miglustat and is intended to treat the neurological symptoms associated with NPC in individuals with an age greater than 2 years. Aqneursa™ has also been approved to be used for the treatment of both adults and children [110,111]. A promising compound, 2-hydroxypropyl-β-cyclodextrin (HP-β-CD) has been shown to effectively transport unesterified cholesterol into the cytoplasm. In animal studies, HP-β-CD has demonstrated a reduction in neurological dysfunction and lipid accumulation, making it a promising drug for the treatment of NPC1 [108].

Progress with viral vectors for NPC1 has been limited due to the large gene size, complicating AAV vector design. Hughes et al. (2023) addressed this by using AAV serotype 9 vectors with a truncated human *NPC1* promoter to stay within packaging limits. This approach showed promising results in mouse models, but further vector optimization is needed before advancing to clinical trials [112]. Another potential drug candidate was developed in a preclinical trial by Jiang et al. (2020), consisting of transferrin receptor-targeted Trojan horse liposomes encapsulating pDNA encoding the *NPC1* gene. This system successfully delivered the pDNA and produced NPC1 mRNA in the spleen, liver, and brain, leading to a reduction in both peripheral organs and the brain. However, this treatment did not extend the lifespan of the animal models, indicating that intervention should begin as early as possible after birth or, if the disease is confirmed in utero, treatment may start before birth [42].

### 3.5. Neurological Disorders

Neurological diseases encompass a range of illnesses affecting the nervous system. These diseases are the leading cause of disability in countries such as the United States, where nearly 1000 neurological disorders impact over 100 million Americans, accounting for 50% of the overall health burden in the country. Significantly, the prevalence of neurological disorders has increased in recent decades, reflecting demographic shifts in both developed and developing nations. The understanding of neurological disorder pathogenesis, from single-gene defects to complex processes, has driven the development of innovative gene-therapy-based treatment strategies [113].

#### 3.5.1. Fabry Disease

Fabry disease (FD) is a multisystemic X-linked lysosomal storage disorder caused by mutations in the *GLA* gene, which encodes the enzyme α-galactosidase A (α-Gal A). Defects in α-Gal A leads to the accumulation of glycosphingolipids in lysosomes, primarily affecting vascular endothelial and smooth muscle cells [43,114]. In Europe, FD prevalence ranges from 1 in 3100 to 1 in 22,000, but, due to the heterogeneity of the clinical phenotypes of this disease, the real prevalence may be higher [115]. The onset of symptoms can be seen from childhood to later stages of life, but the main causes of death in FD patients are cardiac, renal, and cerebrovascular complications [116]. Enzyme replacement therapy has been the standard treatment for FD, but it provides only temporary symptom relief and requires frequent dose administrations, which have a significant impact on the quality of life of these patients [117]. As a result, gene therapy approaches are being explored as alternative solutions for long-term effects.

Current research on FD treatment has primarily focused on gene delivery using viral vectors like lentiviruses and AAVs [118]. In a phase I clinical trial by Khan et al. (2021), five adults with type I FD received autologous CD34+ hematopoietic stem/progenitor cells transduced with lentivirus to express α-Gal A. Within a week, all patients showed near-normal α-Gal A levels without serious side effects, indicating the therapy’s promise. However, concerns remain about long-term risks such as insertional mutagenesis and the limited trial scale, raising questions about broader safety and efficacy in larger patient groups [119]. Specifically for FD nephropathy, Cui et al. (2023) conducted research that utilizes CRISPR technology to suppress A4GALT, leading to the rescue of FD nephropathy phenotype, presenting another possible avenue for gene therapy in FD [44].

Considering this, other non-viral approaches have been explored and implemented. Rodríguez-Castejón et al. (2022) developed a galactomannan-decorated lipid nanoparticle to deliver pDNA encoding α-Gal A, achieving efficient internalization and protein synthesis in liver cell lines without causing significant hemolytic activity or erythrocyte agglutination. In vivo studies in mice have shown that the vector enabled α-Gal A synthesis at clinically relevant levels in the heart and kidneys, the primary organs affected by the Fabry disease, suggesting its potential as an alternative gene therapy for FD [43].

#### 3.5.2. Amyotrophic Lateral Sclerosis

Amyotrophic lateral sclerosis (ALS) is a neurodegenerative disease affecting the central nervous system, characterized by progressive motor function loss and later difficulties with automatic functions like swallowing and breathing. Mutations in over 40 genes, including *C9orf72*, *TARDBP*, *SOD1*, and *FUS*, are linked to ALS, with these genetic changes increasing the risk of developing the disease but not guaranteeing its onset [120]. The heterogeneity of ALS means symptoms and severity depend on the specific mutations involved, but the condition generally leads to motor neuron death, often due to dysfunctional SOD1 enzymes or toxic accumulation of the TDP-43 protein [45]. Familial ALS accounts for 10–15% of cases, while sporadic cases make up the remaining 85%, with an incidence of 1.68–1.89 per 100,000 people [121]. Although ALS remains incurable, ongoing clinical trials are exploring treatments like antibodies, antioxidants, anti-glutamate agents, antisense oligonucleotides, stem cells, and pDNA. Currently, two approved therapies—riluzole and edaravone—help reduce neuron toxicity and slow disease progression [120].

The complex etiology of ALS, involving various gene mutations, poses challenges for applying gene therapy as a treatment. Despite these difficulties, efforts have been made to use viral vector gene therapy, although manufacturing and targeted delivery to the central nervous system (CNS) remain challenging. Notably, studies using AAV vectors to deliver neurotrophic factors such as insulin-like growth factor 1 (IGF-1) and glial cell line-derived neurotrophic factor (GDNF) in mouse models have shown promising results, including increased survival rates and delayed disease onset [122]. In clinical trials, pDNA encoding *VEGF* and hepatocyte growth factor (*HGF*) genes have been explored in phase 2 and phase 1/2 trials, respectively, for ALS treatment. Unfortunately, the development of the *VEGF* pDNA has been halted, and there is limited information available regarding the progress of the *HGF* pDNA [45]. In vivo preclinical trials conducted by Chen et al. (2022) utilized SOD1-ALS mouse models treated with CRISPR gene-editing tools delivered via AAV viral vectors. The study demonstrated a reduction in the mutant huSOD1 protein within the spinal cord and cortex, decreased axonal damage, and preservation of compound muscle action potential throughout the lifespan of the treated mice. Additionally, a significant increase in lifespan was observed: treated SOD1G93A mice lived an average of 110 days longer, while adult mice treated immediately before the symptom onset with a single intrathecal or intravenous injection of the biopharmaceutical enjoyed a lifespan extended by 170 days [46].

#### 3.5.3. Rett Syndrome

Rett syndrome (RTT) is a progressive neurological disorder caused by mutations in the X-linked gene methyl-CpG-binding protein 2 (*MECP2*). This gene encodes a transcriptional regulator that significantly impacts gene expression and neuronal development. MECP2 is often referred to as the “Goldilocks” protein because it requires a specific level of activity for optimal function. RTT results from heterogeneous loss-of-function mutations that lead to insufficient functional protein levels, culminating in the onset of this disorder. Conversely, excessive levels of MECP2 due to locus duplication can lead to conditions such as MECP2 duplication syndrome (MDS), which is associated with severe neurodevelopmental disorders. This duality presents a significant challenge for gene therapy applications, since it is necessary to increase *MECP2* at specific levels to avoid exacerbating problems associated with both underproduction and overproduction of the protein [123]. This disorder primarily affects females, with a prevalence of 1 in 10,000–15,000 live female births, and is characterized by impairments in speech, hand use, and coordination, making communication difficult even without cognitive impairment. Rett syndrome (RTT) progresses through four stages: developmental stagnation, rapid regression, plateau, and motor deterioration. Most treatments focus on symptom relief, with Trofinetide (Daybue^®^, Acadia Pharmaceuticals, San Diego, CA, USA), being the only Food and Drug Administration (FDA)-approved medication specifically for RTT [124,125]. Several research groups have made attempts at conventional gene therapy for hemizygous male mouse models of RTT. Sinnet et al. (2017) successfully rescued normal phenotypes in these mice using AAV vectors to transfer the *MECP2* gene. However, this approach was limited in its preclinical efficacy and safety evaluations, particularly in heterozygous female mice, due to the challenges posed by random X-chromosome inactivation. Additionally, despite the positive outcomes observed in mouse models, there were some concerns about liver toxicity associated with the use of viral vectors [126]. As an alternative, in a preclinical study, Le et al. (2019) created a CRISPR/Cas9-mediated *MECP2* modifications using pDNA encoding the Cas9 protein and the *MECP2* repair template. This system achieved homologous recombination efficiencies of 20–30% in human cell lines and induced pluripotent stem cells [48]. A year later, Croci et al. presented another preclinical application using CRISPR/Cas9 to perform gene editing in the *MECP2* gene. In this study, a human neuronal model derived from reprogrammed patient-derived primary fibroblasts and a set of two pDNA were used. This system resulted in a high editing efficiency, with up to 80% of homologous directed repair, representing a significant improvement over its predecessor [47].

### 3.6. Blood Disorders

Hereditary blood disorders encompass around 5000 conditions caused by mutations in over 200 genes related to blood components like red and white blood cells and platelets. These disorders often result from point mutations in hematopoietic stem cells (HSCs), leading to defective blood cell production. While patients frequently require transfusions, these are limited by donor availability and ongoing treatment needs. Allogeneic HSC transplantation is the only curative option, but it faces challenges with HLA matching and risks such as graft failure and graft-versus-host disease (GVHD). Research is, therefore, focusing on safer ex vivo gene therapy approaches, including autologous transplantation of genetically corrected HSCs and the use of induced pluripotent stem cells (iPSCs) [127].

#### Hemophilia A

Hemophilia A is a rare recessive X-linked disorder, resulting from mutations in the *F8* gene, which encodes the clotting factor VIII. Due to its X-linked inheritance, and because men do not have a second X chromosome to compensate, this condition predominantly affects men, with an incidence rate of 24.6 cases per 100,000 live births. Despite its rarity, hemophilia A poses significant health problems [128]. The mutation causing hemophilia A leads to a deficiency in the clotting factor, resulting in spontaneous or trauma-induced bleeding that can be life-threatening. Management generally requires lifelong clotting factor VIII administration [129]. The monogenic nature of hemophilia A makes it a promising candidate for gene therapy, with significant progress made in recent years. A phase III trial by Ozelo et al. tested valoctocogene roxaparvovec (AAV5-hFVIII-SQ) in men with severe hemophilia A, using an AAV6 vector to deliver factor VIII cDNA with a liver-specific promoter. The study showed an 83.8% reduction in bleeding episodes and increased factor VIII levels, though 16.4% of the patients had adverse effects, such as elevated liver enzymes, headache, and nausea. Further follow-up is needed to evaluate the therapy’s long-term benefits [130]. To develop a potential novel treatment course that targets the core of the disorder, Peng et al. (2009) explored a novel hemophilia A treatment by using pDNA encoding factor VIII in a preclinical trial, achieving long-term factor VIII expression in hemophilia A mice. Multiple injections, combined with anti-CD3 immunomodulators, helped prevent immune responses against factor VIII [50]. In the same year, Miao et al. (2009) used the same pDNA encoding factor VIII, but the mice models received factor-VIII-specific regulatory T cells that significantly reduced the immune response when the pDNA was injected [49].

### 3.7. Muscular Disorders

Muscular disorders are conditions that affect muscle function, causing weakness and reduced mobility. Many are genetic, like muscular dystrophies, and often appear in early childhood, hindering normal developmental milestones due to muscle weakness and delayed motor skills [131]. Although muscular dystrophies constitute a group of more than 50 genetically distinct pathologies, they are grouped into a single disease family that is characterized by progressive muscle degeneration and weakness. Furthermore, the common muscular dystrophies often involve damage to the muscle cell membrane which disrupts homeostasis and leads to secondary effects, such as chronic inflammation and fibrosis [132]. The wide range of muscle disorders often have a severe impact on the quality of life of patients from an early age and are often systemic. Therefore, advances in treatment are very relevant, with gene therapy showing great promise for the treatment of inherited muscular disorders like dystrophies [133].

#### Duchenne Muscular Dystrophy

Duchenne muscular dystrophy (DMD) is a rare, progressive genetic disorder primarily affecting males, caused by mutations in the *DMD* gene that lead to dystrophin deficiency. Without dystrophin, muscles are more vulnerable to damage, resulting in progressive muscle loss, movement difficulties, cardiomyopathy, and, often, respiratory failure. DMD affects fewer than 10 per 100,000 males, with a very low incidence in females [134]. DMD remains an incurable disease primarily managed through symptom relief and prevention, with some therapies existing for particular cases [135]. In 2014, ataluren (Translarna^®^, PTC Therapeutics Limited, Dublin, Ireland) received standard approval by the European Medicines Agency (EMA) as a gene therapy for patients with a non-sense mutation in the dystrophin gene. This therapy is indicated for patients aged two years and older who retain the ability to walk. In a 48-week study, involving 174 patients, ataluren demonstrated a significant benefit, increasing the distance patients could walk by at least 32 m on average. Notably, patients with a progressive decline in walking abilities showed the most substantial improvement, gaining up to 50 m [136]. Two years later, the FDA approved eteplirsen (Exondys 51^®^, Sarepta Therapeutics, Cambridge, MA, USA), the first antisense oligonucleotide designed to modulate splicing for Duchenne muscular dystrophy (DMD) in the United States. This biopharmaceutical is indicated for patients with a mutation in the dystrophin gene amenable to exon 51 skipping. However, its clinical benefits have not yet been fully demonstrated, necessitating further clinical trials to comprehensively assess the therapy’s impact [137,138]. Last year, the FDA approved a novel gene transfer therapy, delandistrogene moxeparvovec-rokl (Elevidys^®^, Sarepta Therapeutics, Cambridge, MA, USA), for the treatment of DMD. The efficacy of Elevidys^®^ was assessed in two double-blind, placebo-controlled studies and two open-label studies, enrolling a total of 218 male patients (including those who received a placebo) with confirmed disease-causing mutations in the *DMD* gene [139]. This therapy utilizes an adeno-associated viral vector to deliver a “micro-dystrophin” recombinant gene, enabling the expression of a smaller yet functional dystrophin protein [140]. In the search for potential treatments, Pini et al. demonstrated that CRISPR gene-editing tools targeting a specific genomic region within a *DMD* duplication could restore endogenous dystrophin production. By comparing continuous and transient nuclease expression, researchers observed equivalent restoration levels, with 50% of dystrophin production achieved in treated myoblasts. This finding suggests that constant Cas9 nuclease expression is unnecessary to target the duplication region of the *DMD* gene effectively [51].

A year later, Maze et al. (2023) developed a dystrophin-encoding plasmid in a preclinical trial that successfully expressed the desired transgene with tissue selectivity, potentially paving the way for novel DMD treatments [33]. This approach addressed the challenge of the large size of the *DMD* gene which makes it difficult to use viral vectors such as AAV, known for their limited packaging capacity. In a recent study, Wang et al. investigated micro-dystrophins, smaller *DMD* gene versions that retain function and fit in AAV vectors, showing promise for gene therapy. However, challenges like systemic dystrophin restoration, immune risks from high vector doses, and limited AAV production suggest the potential benefits of non-viral alternatives [133].

## 4. Plasmid DNA Gene Therapy Challenges

### 4.1. Quality Control

Non-viral gene therapy, particularly pDNA, shows promise as an alternative to viral vectors, addressing issues like immunogenicity, manufacturing, and safety. While pDNA therapy presents its own challenges, particularly in delivery and stability, it is generally seen as a more suitable option for gene therapy. Effective pDNA delivery depends on stable systems that protect genetic material across biological barriers. For certain tissues, like the CNS, more sophisticated delivery methods are needed to cross the blood–brain barrier and achieving high transfection efficiency remains difficult due to the large size of pDNA [27].

In addition to technical challenges, significant regulatory hurdles must be addressed to ensure that therapeutic agents adhere to established standards. Regulatory bodies, including the FDA and EMA, have defined specific criteria for pDNA products, emphasizing high standards of purity, biological activity, and stability. Ensuring that pDNA remains biologically active and stable during the manufacturing and purification processes is essential for compliance with regulatory standards. Regulatory preference is often given to the supercoiled (sc) isoform of pDNA, as it remains structurally intact, thus preserving its biological function. Furthermore, stringent environmental regulations, such as those controlling temperature, are enforced to enhance pDNA stability. The pDNA manufacturing process must align with current good manufacturing practices (cGMP) and undergo extensive quality assurance procedures, ensuring thorough purification and effective removal of contaminants [141,142]. Purifying pDNA for applications in gene therapies involves a range of challenges and trade-offs that require strategic approaches to ensure effectiveness and safety. As demand for these biomedical solutions continues to rise, there is a pressing need to develop purification processes that are not only efficient and scalable but also cost-effective and compliant with stringent regulatory standards. Chromatography is crucial for large-scale pDNA production [143]. Recent advancements, such as the use of monolithic supports and novel resins, have improved binding capacities and processing times, making chromatography more suitable for industrial-scale purification [144]. Achieving these goals is essential for supporting large-scale production and meeting the increasing market demands for high-quality pDNA in clinical applications. Therefore, pDNA products should meet the requirements described in Table 3 to be used for gene therapy.

It has been stablished that a homogeneous preparation of sc pDNA should have a purity greater than 97% to comply with regulatory standards [145]. Such regulations are necessary to ensure that the therapeutic agent has the desired effect and to avoid possible undesired side effects. However, even if the desired purity, activity, and stability of the pDNA are achieved, these properties are useless if pDNA cannot properly reach the target cell or tissue. Therefore, a suitable delivery vehicle becomes a fundamental key to the success of any type of genetic therapy.

### 4.2. pDNA Delivery

#### 4.2.1. Cellular Intake

When dealing with pDNA, most recent advances have favored the use of nanoparticles for drug delivery and, in particular, for gene therapy [146]. There are many characteristics that contribute to a higher efficiency for gene therapy in nanocarriers, including their specific chemical composition. The nature of the different chemical compositions influences how well the carrier interacts with biological systems and their stability in circulation, and it is directly related to possible surface modifications, like the addition of PEG. This modification can prolong the circulation time of nanoparticles in the bloodstream by inhibiting their recognition phagocytosis by the mononuclear phagocytic system, preventing a premature clearance of the therapeutic agent from the body. Furthermore, the size and composition of the carrier have a huge impact on their efficiency. Smaller nanoparticles tend to have better clearance rates from plasma, facilitating their entry into cells compared to larger ones. In addition, these smaller nanoparticles can more easily cross endothelial barriers, a phenomenon which is very relevant when targeting the CNS. In contrast, decreasing the size of a carrier can affect its carrying capacity. Nevertheless, smaller nanoparticles, in the range of 20 nm, can enter cells without relying solely on endocytic mechanisms, improving their efficacy. Overall, the size of nanoparticles used for drug delivery ranges between 10 and 100 nm. Carrier charge can also affect cellular uptake; positively charged nanoparticles can facilitate a better endosomal scape, allowing more effective drug delivery. Furthermore, the materials used to create nanocarriers must be biocompatible and biodegradable to avoid toxicity and adverse effects. Biodegradable polymers such as poly(DL-lactide-co-glycolide) (PLGA) are commonly used, as they can be safely degraded in the body and release their therapeutic agent [147].

#### 4.2.2. Intranuclear Transport

Once the pDNA is present in the cytoplasm, traversing the nuclear envelope becomes one of the most critical bottlenecks for DNA-based biotherapeutics to achieve their therapeutic effect. This step is essential because the pDNA must reach the cellular machinery within the nucleus to enable target gene expression and elicit the intended therapeutic outcome [148]. pDNA can enter the nucleus with the assistance of DNA nuclear targeting sequences (DTS), such as the simian virus 40 (SV40) promoter sequence, which is often incorporated into pDNA constructs, as a standard sequence promoter or as an enhanced version of the SV40 sequence, inducing a much higher gene expression [149]. These sequences are recognized by specific transcription factors (TFs), triggering importin-mediated transport. This mechanism facilitates the movement of pDNA from the cytoplasm, through the nuclear pore complex (NPC), and into the nucleus [150]. Moreover, tissue-specific DTS have been developed, such as the smooth muscle γ-actin promoter, which mediates NPC transport specifically in smooth muscle tissue [151]. From another perspective, during mitosis, the temporary disassembly of the nuclear envelope provides an opportunity for pDNA mobilization into the nucleus of nascent cells [152]. However, this approach is limited in vivo, as many cells are non-dividing (postmitotic) or require extended periods for cell turnover [153]. A promising application to enhance the transport of foreign DNA through the NPC was presented by Liedl et al. (2023). Their approach involves the use of DNA origami—a three-dimensional complex consisting of target DNA, multiple SV40-derived DTS, and a mammalian-cell reporter gene. This DNA origami demonstrated the ability to traverse the NPC, showing that DTS sequences on the surface of the DNA origami are sufficient to leverage importin-mediated transport of macromolecules through nuclear pores. Further research is needed to explore additional potential complexes of target DNA and DTS sequences. Efforts should focus on improving the binding affinities between these complexes and cellular proteins responsible for importin-mediated transportation, as well as broadening their applications to accommodate various target sequences and sequence sizes [148].

## 5. Conclusions

In this review, we discuss the applications of pre-clinical (in vitro and in vivo) and clinical pDNA-based potential novel therapeutics for several rare monogenic diseases. These disorders have been receiving growing attention from both academia and the pharmaceutical industry, with academic institutions and private investors representing the primary contributors to research and development efforts for new therapies [154].

Orphan-disease-directed novel therapeutics have been based on two different types of vectors, viral and non-viral vectors, to deliver wild-type genes, silencing sequences, or gene-editing systems. Among these vectors that can be applied to this category of disorders, pDNA presents itself as an interesting and versatile option, as it can be applied to a variety of different diseases, with varying sizes of the gene of interest. The ability to use large-size genes and incorporate them into the plasmid sequence is an advantage compared to viral vectors, although an increase in plasmid size risks decreasing delivery and incorporation efficiencies. This loss in delivery can be mitigated by combining the pDNA vector with a tailored delivery system, such as lipid nanoparticles and micelles, which possess ligands that bind to specific membrane receptors present on the surface of the target cells. Furthermore, to reduce the risk of inflammation associated with the application of a pDNA-based therapeutic, the removal of CpG sequences decreases immunogenicity while simultaneously enhancing the sustainability of transgene expression. However, pDNA-based therapies face challenges with long-term expression, a notable disadvantage compared to viral vectors, highlighting the need for improvements in expression cassettes. An alternative to pDNA could be minicircle DNA (mcDNA), as it allows the delivery of large genes of interest without compromising transfection efficiency. Additionally, the development of novel delivery systems and ligands may offer promising solutions to pDNA transfection challenges. Identifying key membrane molecules overexpressed in target tissues and cells could serve as a crucial starting point for designing more efficient delivery methods. The development of new cellular and animal models for rare monogenic diseases is another critical area that requires special attention. These models are essential for overcoming one of the major bottlenecks in the development of novel therapeutics for monogenic orphan diseases, as they are crucial for progressing to human clinical trials.

## Figures and Tables

**Table 1:** Comparison of viral and non-viral vectors [25,26].

	Non-Viral Vectors	Viral Vectors
**Transgene expression**	Transient expression	Transient for long-term expression
**Efficiency**	Lower transfection efficiency	Higher transduction efficiency
**Cargo capacity**	Medium to large	Small to large
**Insertional mutagenesis**	Reduced risk	Risk for some viral vectors
**Immunogenicity**	Low	Possible
**Specificity**	High	Low specificity for some cell types
**Requires custom delivery method**	Yes	No
**Production process**	Versatile and cost-effective	Complex and costly

**Table 2:** Overview of studies of non-viral gene therapy for monogenic diseases.

Type of Disease	Disease	Conventional Treatment	Active Substance in Gene Therapy	Encoded Gene/ Protein	Delivery	Type of Studies	Reference
Ocular Disorders	Retinitis pigmentosa	UV-protecting glasses, vitamin supplements, and retinoids	Plasmid	CRISPR-Cas9	-	In vitro trials	[29]
	Retinitis pigmentosa/ macular telangiectasia type 2		Encapsulated human retinal pigment epithelial cell line transfected with a plasmid vector	CNTF	Encapsulated Cell Technology^®^	Phase II (NCT00447980)	[30]
	Stagardt disease	No FDA- approved therapy	Plasmid	ABCA4	ECO/DNA self-assembled nanoparticles	In vitro and in vivo trials	[31]
					PEG-ECO/DNA nanoparticles	In vivo trials	[32]
Respiratory disorders	Cystic fibrosis	CFTR modulator and antibody-based drugs	Plasmid	CFTR	Naked DNA	In vitro trials	[33]
			Plasmid expressing Cas9	CRISPR-Cas9	-	In vitro trials	[34]
			Plasmid	CFTR	Cationic liposome	Phase IIb (NCT01621867)	[35]
			Plasmid	CRISPR-Cas9	-	In vitro trials	[36]
	Primary ciliary dyskinesia	-	mcDNA	DNH5	Liposome	In vivo trial	[37]
	AAT deficiency	Plasma- purified AAT intravenous augmentation therapy	Plasmid	AAT	Nasal mucosa	Phase II (NCT02001688)	[38]
				CRISPR-Cas9	-	In vivo trials	[39]
Skin Disorders	Recessive dystrophic epidermolysis bullosa	Gene therapy w/ herpes-simplex virus vector	mcDNA	COL7A1	Poly(β-amino ester) polymeric nanoparticles	In vitro trials	[40]
			Plasmid	CRISPR-Cas9	-	Ex vivo trials	[41]
Metabolic Disorder	Niemann–Pick C1	Miglustat and HP-β-CD	Plasmid	Human NPC1 open reading frame	Pegylated liposomes	In vivo trials	[42]
Neurological Disorders	Fabry disease	Enzyme replacement therapy	Plasmid	α-Gal A	Galactomannan-decorated lipidic nanocarrier	In vitro and in vivo trials	[43]
				CRISPR-Cas9	-	In vitro trials	[44]
	Amyotrophic lateral sclerosis	Riluzole, edaravone, sodium, phenylbutyrate, and taurursodiol	Plasmid	ZFP-TF that upregulates endogenous VEGF-A	Intramuscular	Phase II (NCT00748501)	[43,45]
				HGF		Phase I/II (NCT02039401)	[45]
				CRISPR-Cas9	-	In vivo trials	[46]
	Rett syndrome	Trofinetide	Plasmid	CRISPR-Cas9 to edit MECP2	-	In vitro trials	[47,48]
Blood Disorder	Hemophilia A	Protein replacement therapy	Plasmid	Factor VIII	-	In vitro and in vivo trials	[49,50]
Muscular Disorder	Duchenne muscular dystrophy	Ataluren, Eteplirsen, delandistrogene moxeparvocec-rokl, and corticosteroids	Plasmid	CRISPR-Cas9	-	In vitro trials	[51]
				Dystrophin	Naked DNA	In vitro trials	[33]

Abbreviations: α-Gal A—α-galactosidase A; AAT—a-1 antitrypsin, ABCA4—ATP-binding cassette subfamily A member 4; COL7A1—collagen type VII alpha 1 chain; CFTR—cystic fibrosis transmembrane conductance regulator; CNTF—ciliary neurotrophic factor; CpG—5′—C-phosphate-G-3′; CRISPR-Cas9—clustered regularly interspaced palindromic repeats-associated protein 9; DNAH5—dynein axonemal heavy chain 5; DNA—deoxyribonucleic acid; ECO—(1-aminoethyl) iminobis [N-(oleicylcysteinyl-1-amino-ethyl)propionamide]; eGFP—enhanced green fluorescence protein; food and drug administration (FDA); HGF—hepatocyte growth factor; hIgG1—human immunoglobin G1; 2-HP-β-CD—hydroxylpropyl-β-cyclodextrin; hTNFR-Is—human tumor necrosis factor-alpha-soluble receptor I; mcDNA—minicircle DNA; MECP2—methyl-CpG-binding protein 2; NPC1—Niemann–Pick disease type C1; PEG—polyethylene glycol; pDNA—plasmid DNA; TNF-α—tumor necrosis factor alfa; VEGF—vascular endothelial growth factor; ZFP-TF—zinc finger protein transcription factor.

**Table 3:** Overview of the impurities associated with the production of pDNA [27,141,142].

Impurity	Cut-Off	Detection Method
Genomic DNA	<2 ng/µg pDNA	qPCR
Host protein	Undetectable	Microassay w/BSA calibration curve
RNA	Undetectable	Agarose gel electrophoresis
Open circular and linear isoforms	<3% of total pDNA	Agarose gel electrophoresis, analytical chromatography
Endotoxins	<0.1 EU/µg pDNA	Limulus amebocyte lysate (LAL) assay

## Abbreviations

α-Gal A	Alpha-galactosidase A
AAT	Alpha-1 antitrypsin
AAV	Adeno-associated virus
ABC	ATP-binding cassette
ABCA4	ATP-binding cassette subfamily A member 4
ALS	Amyotrophic lateral sclerosis
BBB	Blood–brain barrier
COL7	Type VII collagen
COL7A1	Collagen type VII alpha 1 chain
CF	Cystic fibrosis
CFTR	Cystic fibrosis transmembrane conductance regulator
CNTF	Ciliary neurotrophic factor
CNS	Central nervous system
CpG	5′—C—phosphate—G—3′
CRISPR-Cas9	Clustered regularly interspaced palindromic repeats-associated protein 9
DMD	Duchenne muscular dystrophy
DNAH5	Dynein axonemal heavy chain 5
DNA	Deoxyribonucleic acid
DTS	DNA nuclear targeting sequence
ECO	(1-aminoethyl) iminobis [N-(oleicylcysteinyl-1-amino-ethyl)propionamide]
eGFP	Enhanced green fluorescence protein
EMA	European Medicines Agency
FD	Fabry disease
FDA	Food and drug administration
FEV	Forced expiratory volume
GDNF	Glial cell line-derived neurotrophic factor
GND	Gold nanodiamond
GNR	Gold nanorod
gRNA	Guide RNA
GVHD	Graft-versus-host disease
HA	Hyaluronic acid
HGF	Hepatocyte growth factor
hIgG1	Human immunoglobin G1
HLA	Human leukocyte antigen
HP-β-CD	Hydroxypropyl-β-cyclodextrin
HSCs	Hematopoietic stem cells
HSP-1	Herpes simplex virus 1
hTNFR-Is	Human tumor necrosis factor-alpha-soluble receptor I
IGF-1	Insulin-like growth factor 1
IMDs	Inherited metabolic disorders
INP	Inorganic nanoparticle
iPSCs	Induced pluripotent stem cells
mcDNA	Minicircle DNA
MDS	MECP2 duplication syndrome
MECP2	Methyl-CpG-binding protein 2
mRNA	Messenger RNA
MTT2	Macular telangiectasia type 2
ND	Nanodiamond
NPC	Nuclear pore complex
NPC1	Niemann–Pick disease type C1
PCD	Primary ciliary dyskinesia
pDNA	Plasmid DNA
PEG	Polyethylene glycol
PLGA	poly(DL-lactide-co-glycolide)
PLNP	Polymeric lipid nanoparticle
RDEB	Recessive dystrophic epidermolysis bullosa
RGCs	Retinal ganglion cells
RISC	RNA-induced silencing complex ribonuclease
RNA	Ribonucleic acid
RP	Retinitis pigmentosa
RTT	Rette syndrome
sc pDNA	Supercoiled pDNA
siRNA	Small interfering RNA
SLNP	Solid lipid nanoparticle
STGD	Stargardt disease
SV40	Simian virus 40 promoter sequence
TF	Transcription factors
TNF-α	Tumor necrosis factor alfa
VEGF	Vascular endothelial growth factor
